# “Picking up the pieces”—Meanings of receiving home nursing care when being old and living with advanced cancer in a rural area

**DOI:** 10.3402/qhw.v10.28382

**Published:** 2015-09-10

**Authors:** Siri Andreassen Devik, Ove Hellzen, Ingela Enmarker

**Affiliations:** 1 Centre of Care Research, Mid-Norway, Steinkjer, Norway; 2 Department of Nursing, Mid-Sweden University, Sundsvall, Sweden; 3 Department of Health Sciences, Nord-Trøndelag University College, Stjørdal, Norway

**Keywords:** Advanced cancer, rural palliative care, homecare, district nursing, old people, identity, place attachment, qualitative research, phenomenological hermeneutics

## Abstract

Rural home nursing care is a neglected area in the research of palliative care offered to older cancer patients. Because access to specialized services is hampered by long distances and fragmented infrastructure, palliative care is often provided through standard home nursing services and delivered by general district nurses. This study aimed to illuminate the lived experience and to interpret the meaning of receiving home nursing care when being old and living with advanced cancer in a rural area in Norway. Narrative interviews were conducted with nine older persons, and a phenomenological hermeneutic approach was used to interpret the meaning of the lived experience. The analysis revealed three themes, each with subthemes: *being content with what one gets*, *falling into place*, and *losing one's place*. The phrase *picking up the pieces* was found useful to sum up the meaning of this lived experience. The three respective themes refer to how the pieces symbolize the remaining parts of life or available services in their environment, and how the older persons may see themselves as pieces or bricks in a puzzle. A strong place attachment (physical insideness, social insideness, and autobiographical insideness) is demonstrated by the informants in this study and suggests that the rural context may provide an advantageous healthcare environment. Its potential to be a source of comfort, security, and identity concurs with cancer patients’ strong desire for being seen as unique persons. The study shows that district nurses play an essential role in the provision of palliative care for older rural patients. However, the therapeutic value of being in one's familiar landscape seems to depend on how homecare nurses manage to locate it and use it in a more or less person-centred manner. Communication skills and attentiveness to psychosocial aspects of patient care stand out as important attributes for nursing in this context.

Because most palliative care patients prefer to remain in their own homes as long as possible (Higginson & Sen-Gupta, 2000), more emphasis is placed on delivering high quality care where people live. In Norway (Holm, 2012), as in the rest of the world (Ramsey & Beesley, 2007; Robinson et al., 2009), long distances, fragmented infrastructure, and shortage of health human resources are well-known barriers that complicate access to specialized palliative care. Palliative patients in this context, especially the older population (Sigurdsson et al., 2009), therefore often only receive standard home nursing care (Goodridge & Duggleby, 2010; Kaasa, Jordhøy, & Haugen, 2007).

Much attention has been given to specialized palliative care services (e.g., multiprofessional teams, specialized palliative care teams), but relatively few studies have examined the context of general district nursing (Burt, Shipman, Addington-Hall, & White, 2008). Burt et al. (2008) found that community nurses identified palliative care with workload stresses in both time and emotion. The nurses perceived that they worked in a pressurized context, characterized by shortage of staff, and lack of time and formal support. Although education regarding physical care such as pain and symptom management is often viewed as the highest need among nurses in this context (Easom, Galatas, & Warda, 2006), psychosocial aspects are perceived as the most challenging (Walshe & Luker, 2010).

Several studies that focus on rural palliative care include data from multiple perspectives, but few aim to directly assess the perspective of the patient (Robinson et al., 2009; Walshe & Luker, 2010). For example, Pesut, Bottorff, and Robinson (2011) included family members, volunteers, and health professionals to gain an understanding of the values informing good rural palliative care. Core values were identified: knowing and being known, being present and available, and community and mutuality as being the foundations for ethically good rural palliative care. These values were highly prized, but each came with a corresponding ethical tension that could result in loss of privacy, high expectations, and caregiver strain. Other findings (Duggleby et al., 2011) also indicate that the rural context has a major impact on community support and healthcare services needed by older persons with advanced diseases. This study was also carried out with a mixed perspective of patients with incurable cancer, bereaved family caregivers, and health professionals.

The unique and direct experience of those who are the recipients of care appears to be under-researched and seems critical to explore. Earlier, Devik, Enmarker, Wiik, and Hellzen (2013) found that older rural palliative cancer patients who lived alone, to a large degree, were left alone in managing their daily lives and in commuting to policlinic care. The absence of home nursing care was striking considering the problems described by these patients, and a suffering dimension was found associated with the insufficient provision of care. In continuation of these findings, and with the aim of increasing knowledge of the rural context, our scope was focused on the experience of receiving home nursing care. In addition, we wanted to explore how the social and physical environments in rural settings may serve as health determinants or how “place of care” influences quality of life. According to Keating (2008), research concerning older adults living in rural areas has not critically addressed how context influences their perceived opportunities and constraints. The aim of this study was therefore to illuminate and interpret the meaning of receiving home nursing care when being old and living with advanced cancer in a rural area in Norway.

## Method

### Design

This study adopted a qualitative design placed within the tradition of phenomenological hermeneutics (Ricoeur, 1976) described by Lindseth and Norberg (2004). Based upon a lifeworld approach (Husserl, 1970), narrative interviews were conducted to gain insight into the participants’ lived experiences of receiving home nursing care in this context.

### Research context and recruitment procedure

Persons were considered eligible if they were aged 65 years or older, diagnosed with advanced cancer (curable treatment was no longer an option), and receiving home nursing care in a rural area. Definitions of rurality seem to differ markedly between countries and research contexts (Steinhaeuser, Otto, Goetz, Szecsenyi, & Joos, 2014). A meaningful definition of rural, according to the Norwegian context, is a territorial area with scattered settlements, some distance from a densely populated area, and where natural resources are the basis for industry and outdoor activity (Haugen & Stræte, 2011).

A convenient sample was recruited depending on willingness and on each person's physical and psychological suitability. Oncology nurses, or nurses with specialized training to care for persons with cancer, in a district covering 23 municipalities were informed about the study in meetings with the researcher. Both oral and written information were provided. These nurses judged whether each person's health status permitted participation and eligible candidates were informed both orally and by a letter. Persons who wished to participate mailed their consent directly to the researcher. The researcher then contacted the participant by phone and practical arrangements were agreed upon.

### Participants

From January 2013 to April 2014, nine participants (five women and four men) gave their consent. Their ages ranged from 71 to 92 years (mean 80.2 years), and they resided in rural areas where the number of inhabitants varied between 268 and 5089 (mean 5 inhabitants/km^2^). The amount of time they had lived with cancer and the type of diagnosis varied. Six of the participants lived alone. Characteristics of the participants are shown in Table I.

**Table I:** Overview of the informants.

Sex	Age	Living arrangements	Type of homecare services
Female	74	With husband in sheltered housing	Safety alarm Personal hygiene Pill dispenser
Male	76	Alone in own house	Daily medication
Male	77	Alone in own house	Pill dispenser
Female	75	Alone in sheltered housing	Daily medication
Male	85	With wife in own house	Pill dispenser
Female	71	Alone in own house	Pill dispenser
Female	88	Alone in sheltered housing	Help with getting dressed, pill dispenser
Male	84	With wife in own house	Can call for visits/supervision from oncology nurse when needed
Female	92	Alone in sheltered housing	Personal hygiene, help with getting dressed, daily medication

### Data collection

The first author collected the data by using a narrative interview technique (Mishler, 1986). The interviews took form as dialogues between the respondents and the researcher.

Open-ended questions were used to encourage the respondents to talk about their personal experiences of receiving home nursing care when living with advanced cancer in this rural context. The following primary questions guided the conversation: Can you please tell me about the care you receive in your daily life and how you experience it? Can you please tell me about an experience of good care and not-so-good care that you have experienced?

The researcher tried to interrupt as little as possible, only commenting to understand their stories and to expand their feelings and meanings. Questions that were asked to follow up participants’ comments included: What happened then? Can you please tell me more? How did you feel? Having the opportunity to tell their stories freely emerged as very important to the respondents. The narratives, therefore, took form as life stories rather than focusing only on each person's present situation. The interviews took place in the participants’ homes and lasted from 52 to 95 min. The interviews were audiotaped and transcribed verbatim.

### Data analysis

A phenomenological hermeneutical approach (Lindseth & Norberg, 2004) was taken to interpret the meaning of this lived experience. The aim of this analysis is to unveil the meaning of the text message and the possible world that the transcripts reveal. The interpretation goes beyond what the text actually says to what it talks about (Ricoeur, 1976), and the objective is never to give a direct reproduction of what the respondent really meant. The process consists of three phases: a naïve understanding, a structural analysis, and finally a comprehensive understanding. The method combines phenomenological description and hermeneutical interpretation. In the first phase, the text was read several times to achieve an open and initial (naïve) understanding of the text (from all interviews) as a whole. We attempted to reach an understanding of the immediate impression of receiving home nursing care in this context. This understanding was regarded as a first conjecture, which had to be validated or invalidated by the structural analysis. The structural analysis started by scrutinizing the text to identify meaning units. These meaning units were then condensed, abstracted, and interpreted to be part of patterns that gave meaning to subthemes and themes. During the structural analysis, the meaning units were considered as independently as possible from their context in the text. Then, the subthemes and themes were reflected upon in relation to the naïve reading. Based upon the validated initial reading and the structural analysis, an interpreted whole was formulated. In this last phase, we aimed to reach a comprehensive understanding by seeing the whole in light of its parts and the parts in light of the whole. The authors’ horizons and theoretical framework, as well as the findings from the previous phases, were taken into account.

## Ethical considerations

The regional research ethics committee of south-east Norway approved the study (No. 2012/1145). The participants received oral and written information about the purpose and procedure of the study. They were assured confidentiality, and all participants gave their written consent.

## Findings

### Naïve understanding

Receiving home nursing care in this situation means to continue a familiar and meaningful life. Having the opportunity to remain in one's home is highly appreciated. Here, all requisites are known, both inside and outside the house. Memories live in the walls, and in the surrounding nature, and even within people in the surroundings (family, neighbours, and nurses). They know who you are and where you have been. However, receiving home nursing care also means making adjustments and accepting the prevailing conditions. Being a patient often means being restricted and having to relinquish control and expectations. It means to be satisfied with what one is offered. It also means to connect and interact differently with different nurses. Some are inviting, personal, and willing to sit down and talk for a while. Others are distant and busy with the physical “doing” of the job.

### Structural analysis

The structural analysis revealed three themes and six subthemes. An overview of the analysis is shown in Table II.

**Table II:** Overview and examples from the structural analysis—from quotations from the text to condensed meaning and to sub- themes and themes.

Interview text	Condensed meaning unit	Subtheme	Theme
I have always lived here. I don't think about the distances, really. Of course, it takes the time it takes … but our ambulance personnel is good	Being aware of the consequences of rural living	Being realistic	Being content with what one gets
However … I am doing fine … They are all so kind to me and I get tasty meals … I have surely nothing to complaint about	Focusing on gratitude	Being indulgent	Being content with what one gets
I have built this house by myself. If you look out the window you can see our home when we were newly married … that was my grandfather's farm. It was a small farm … we had just a few cows and some pigs, which was common in those days	Being deeply rooted in this place	Belonging here	Falling into place
There are different nurses … there is one who I call *Mum* … she is so nice and helpful. It's good to meet somebody you connect with. We share the same interest for patchwork	Being included	Being confirmed	Falling into place
They couldn't give me a safety alarm. They said It was a technical problem, so I have to use my mobile … But, much has to work out if their assistance is to arrive the minute I need it. It depends on the time of the day and their staff ….	Adjusting to the actual facts	Being restricted	Losing one's place
They are always in a hurry. They have so many to help … and I am only one …	Being one out of many in need of help	Being negligible	Losing one's place

#### Being content with what one gets

Contentedness is found to be a fundamental attitude among the informants. This attitude covers everything from realism and genuine satisfaction to tolerance and even dissatisfaction. However, they seem to have reconciled with the “pieces” that are left.

#### Being realistic

A realistic mentality is characteristic for all the informants as they start to narrate. One of the women says: “Mostly, I think about my past life … I don't know what lies ahead of me. You just have to take one day at a time”.

None of the informants considers his/her rural living to be a disadvantage. Staying at home is highly valued, and the distance to hospital does not provoke anxiety. Rural living is something they are used to, and what it brings with it is taken for granted. They are fully aware of the time it will take them to get to hospital and that an ambulance may be hours away in case of an emergency.

They are also understanding about the delivery of homecare services. One of the women has a safety alarm, but she often has to wait for the nurse to come. She says:

They don't come so fast, always … It depends on … they have so many to look after, you know. Therefore, I often have to wait, but they try to be here as quickly as they can. I have good care.

All informants have undergone periods when their illnesses and symptoms have required acute and intensive care. Now, however, most of them are more stabilized and experience that services from the homecare nurses are reduced. One of the men says:

They used to come several times a day in the beginning … but after my last hospital admission, they haven't been here so much. I guess they are busy … but I accept that I am not given such high priority anymore.

#### Being indulgent

Being indulgent is another way of seeing how the participants bear with a difficult situation. As long as their condition is stable, or does not worsen, life is bearable. Making life bearable seems to be an active process demonstrated in the ways that unfavourable things are smoothed over. Several times, the informants started talking about physical complaints or moments when professionals have been less than forthcoming. Nevertheless, they end their sentences by brushing things aside. For example, one of the men dislikes that some of the nurses walk into his house with their shoes on. He says:

Her shoes were wet and dirty, and she left footprints all over the carpets, so I asked her if she found it too bothersome to leave her shoes in the hall. No, she said … but the same thing happened every time … but I know how it is, because I used to work as a caregiver myself, and sometimes I visited homes where I preferred walking with my boots on. However, I feel that I can be honest, I believe I can …

One of the women talks about a time when she was very sick and all her children were gathered to be informed about her situation: “But to me they told nothing … but I didn't ask either. After all there isn't much to ask about. You just understand it … and the most important thing is to be without pain”.

#### Falling into place

Falling into place incorporates a sense of belonging to home, implicit both to place and to people. It refers to a kind of property—of being in one's legitimate place.

#### Belonging here

The narratives are characterized by a strong attachment to home and place, both in a geographical and a social sense. Above all, family members provide the most significant feelings of having established roots here. Although large geographical distances, hamper their opportunities tospend time with their children and grandchildren as often as they would like, they maintain regular contact by phone. Family pictures seem to be all over their homes, and memories, good and bad, live in the walls. Belonging to the family means being loved and being looked after. One of the men says: “My oldest daughter, she is a nurse. I can ask her about everything. Man, that girl watches over her father! (Laughs)”.

Most of the informants have lived their whole life in the same rural district. Memories of the landscape, environment, and people they once knew often appear in their stories. These memories are frequently visualized (for the interviewer) by drawing attention to the landscape outside the window. Sometimes these memories are expressed with sorrow and other times with laughter. Besides instilling feelings of fitting in, these memories provide confidence—this place and these people will be there for me. One of the men who lives alone demonstrates this trust:

The homecare nurses are stationed 40 kilometres from here, so they can't be here in a minute … But there are three nurses living in this village … so I guess one of them would come if anything happened.

In addition, most nurses in these small communities are familiar to the participants; sometimes they are even relatives. One of the men says: “I know all the nurses – nobody is a stranger. We have this sort of overview out here”.

Another man adds: “The solidarity is great here … we observe each other … and take care of each other … That's just the way it is!”

#### Being confirmed

Although the nurses in general are highly appreciated and viewed as kind and helpful, a higher value is set on some of them. Ability to show a genuine and empathic interest in the patients as persons is a common feature of the appreciated nurses. Technical skills or special competences receive little attention or approval. Instead, personal qualities, such as having a sense of humour or generosity exceeding, what can be expected are often highlighted.

One woman says:

The nurses are different – indeed, they are. Some of them just do it, and then there is nothing more. But when X (the name of one of the nurses) arrives … she dances through the door, you know (laughs) and cheer me up … We have a good connection. It means a lot to me. She is more than a nurse … She is a person.

The interview situation provokes a landslide of old stories and anecdotes from their past life. Having visits from someone willing to listen appears to be a rare thing. One of the women says:

It is usually a quick visit when they come with my pill dispenser every other week … They can sit down a while if I ask them, but I try not to … They are allowed to leave when they have done their job. When I had a wound they spent more time …// But some of them have time to sit and chat … some are able to put themselves in another's place.

#### Losing one's place

Receiving home nursing care acquires accommodation to other persons (nurses) and to the system organizing the care. Daily activities are now under the control of others and one has to make oneself available whenever it suits the system. The older persons often find their actions restricted and individual preferences disregarded.

#### Being restricted

Except from one patient, who receives visits from a specialist nurse (oncology nurse), all informants receive ordinary home nursing care. This may involve the delivery of a pill dispenser, help with personal hygiene, injections, or assistance with getting up in the morning or going to bed in the evening. Daily life appears somewhat empty, divided into hours for meals, medication, and rest in bed. Being in the hands of others is reassuring and unsatisfactory at the same time.

Answering the question of what her day is like, one of the women says:

Well, there isn't much really. I sit here … and then I get the insulin-injections before every meal … Days pass, strange enough, one like the other. Time feels long … But I enjoy listening to the radio. // I have to go to bed at nine o'clock; they have to help me before the night nurse arrives … before their shift ends. When I was still able to take care of myself, I used to get visits from my son and daughter-in-law. We could sit and chat the whole evening … We can't do that anymore.

Being patient and having to wait can also be restricting. One woman says: “Much time is spent waiting, and sometimes you feel that time is wasted … There might be other things you would rather like to do”.

#### Being negligible

Being a care recipient also means being one of the groups needing help. Having the understanding of oneself as being a number in a group may undermine feelings of uniqueness and individuality. Bearing in mind that there are other patients waiting or demanding attention, the informants seem to have reconciled with being just a recipient.

One of the women says: “They (the nurses) come every time they have a task or if I call them. They have many to help, and I am only one. They rarely have time to sit and chat with me”.

The same woman also talks about arranged activities for older people at a nursing home nearby: “No, I haven't been attending that … As I have poor eyesight, there are no activities for me”.

Several of the informants express their longing for something extraordinary to happen, for example, to get an opportunity to spend time doing something they would really like to do. A woman says: “A good day is when something happens, other than what we do every day … something that isn't already organized”.

### Comprehensive understanding

This study elucidated the meaning of receiving home nursing care when being an older person living with advanced cancer in a rural area. The phrase *picking up the pieces* is found to be a useful metaphor for displaying the multidimensional meaning of this lived experience. In one dimension, the meaning of this is seen as the struggle to be satisfied with the pieces life has left—accepting realities and valuing and making use of available resources in one's environment. Another dimension points to the realization of oneself as a piece of the puzzle. Being a piece of the puzzle symbolizes one's belonging and significance in history and place, but it may also mean that one holds up a minor or trivial position among many other “bricks”. The value of this brick, or the self, seems highly reliant upon attachment to the home and the rural context. Their feelings of falling into place—of being inside physically, socially, and autobiographically—facilitate feelings of control, security, and a positive sense of self (Golant, 1984; Rowles, 1990). Homecare nurses play an important role in allowing these older persons to remain in their environment throughout the course of illness. Nurses who listen to and confirm the “brick” being in its rightful place strengthen feelings of insideness and of being worthy.

## Discussion

The metaphor *picking up the pieces* was, in this study, found to be useful for illuminating the meaning of receiving home nursing care when being old and living with advanced cancer in a rural area. The three respective themes refer to how pieces symbolize the remaining parts of life or available services in their environment, and how the older persons may see themselves as pieces or bricks in a puzzle. Following this thread, the puzzle is viewed as the context in scope—the rural area.

The significance of the context is evident in each of the three identified themes. As both a limitation and a benefit, rural living highly influences the meaning of receiving home nursing care for the participants in this study.

Different scholars have linked places and their attendant meanings to older persons’ sense of self, independence, and search for meaning in life-course events (Rowles, 1983; Rubinstein & Parmelee, 1992; Wahl & Lange, 2006; Wiles, 2005). By using the concept of *insideness*, Rowles (1983, 1990) has described place attachment along three dimensions: physical, social, and autobiographical. The first dimension is a sense of being physically familiar and entwined with the environment. After long-time residency, people inhabit routines and have environmental mastery in their homes and communities. The social insideness, then, originates not only from the everyday social exchange with family and friends but also from the perception of being well-known and knowing others. Autobiographical insideness is thought to be the most relevant describing older persons’ attachment to place, because it is embedded in memories. Over a lifetime, individuals collect experiences from different places whereby places become referents to memories, or landscapes of memories, that provide a sense of identity and are an ever-present source of reinforcement for a biography interpreted from the retrospective vista of a life review (Rowles, 1983). Rowles (1993) found that rural seniors develop a deep attachment to their homes, especially in late life and with long time residency. Through this attachment, they gain a source of identity, refuge, and comfort, and thus wish to age in place.

The theme *being content with what one gets* refers to a generally tolerant and modest attitude that prevails in all the narratives. Moreover, it demonstrates a clear wish to age in place. The scarcity of resources, in terms of services, equipment, and competence, is a recognized impediment in rural healthcare (Madigan, Wiencek, & Vander Schrier, 2011; Robinson et al., 2009), and earlier research on remote cancer patients suggests a discrepancy between these patients’ needs for support and how those needs are met (Davis, Williams, Redman, White, & King, 2003; McGrath, 2001; Wilkes, White, Mohan, & Beale, 2006). In the present study, the older persons make no point about rural living being detrimental when it comes to the delivery of home nursing services. Neither deficient services nor anxiety over not receiving acute assistance seems to give rise to concern. In a review, Butow et al. (2012) showed that most rural cancer patients preferred to stay at home, despite worse health-related outcomes and higher needs in the domain of daily living, compared to their urban counterparts. Limited access to services, a self-sufficient life style, and a stoical and reluctant help-seeking attitude were suggested as explanations. Other studies also imply that rural cancer patients demonstrate strong stoicism, underrate their needs, and that their views are characterized by realism (Davis et al., 2003; Devik, Enmarker, Wiik, et al., 2013; Gunn, Turnbull, McWha, Davies, & Olver, 2013; Pesut, Robinson, Bottorff, Fyles, & Broughton, 2010). The realistic and indulgent attitude found in the present study adds to previous findings. Claims and expectations are seldom made explicit, and experiences of not-so-good care are smoothed over. Picking up the options that are actually offered emerges as a natural attitude instead of being an adjustment requiring sacrifice. These findings may argue in favour of a prevailing rural attitude, but there may be other accounts as well.

Older cancer patients are reported to be more satisfied with life than younger patients (Michelson, Bolund, Nilsson, & Brandberg, 2000) and a longer life, in general, may achieve more strength to face the challenges caused by life-threatening illness (De Haes & Knippenberg, 1995).

From another perspective, lack of self-assertive behaviour and contentedness can be explained by strong informal support structures (e.g., friends and family) that may exist in rural communities (Duggleby et al., 2011; White et al., 2011). Being a part of the community's social fabric (*social insideness*, cf. Rowles), residents may earn social capital that they can draw on in the form of support from other residents. It becomes clear that the older persons believe that they will receive help from their community if needed. The environmental interaction and the mobilization of developmental resources are dependent on having this belief (Cookman, 2005).

Rural living means being in one's habitat, which seems to have an intrinsic value that may outweigh possible disadvantages.

The theme *falling into place* condenses the meaning of being in one's element. Inherent in this theme, all three components of place attachment (defined by Rowles) can be found. Being at home is like a brick being in the right place: this is my land and these are my people. Their feelings of belonging here seem to be confirmed by significant others (family, friends, and sometimes nurses) and by engaging in recalling memories of life events. It is strongly related to how the place confirms their identity and makes sense to the life that is lived (*autobiographical insideness*, cf. Rowles). Studies have shown that old people with strong ties to place are also reported to feel more secure, in control, and to have a positive sense of self (Rowles, 1993; Rubinstein & Parmelee, 1992; Sugihara & Evans, 2000). Remaining at home may be significant in being the one constant in a life situation threatened by losses. However, the feeling of being attached to home and place is, if not reliant on, at least strengthened by individual nurses who are obliging and open for a personal relationship.

All our informants longed to talk with someone and to connect with someone. This is in line with Kendall et al. (2006), who showed that cancer patients receiving primary care most value the ongoing support—that is, issues that focus on process and relationships.

Previous research (Devik, Enmarker, & Hellzen, 2013) has shown that community nurses may have trouble balancing their personal emotions and with being what they envision as professional when caring for terminally ill persons who live at home. The nurses in the referred study experienced that patients’ expressions left them with feelings that affected their approach and their actions in terms of being more or less generous. Their understanding of being professional, within the ideal of providing equal care, was deeply challenged. The present study allows the picture to be presented from the opposite side. Like the nurses, the patients perceive expressions from their nurses differently and sense that some nurses more than others emerge as personable and compassionate. Through seemingly small gestures, and not necessarily by exercising special practical competence or skills, some nurses are able to affirm that the older persons are in their rightful places. These nurses are willing to spend a little extra time on listening to stories and getting to know the person behind the story. Person-centred and relational care is seen as synonymous with the best quality of care and as essential in palliative care (Mok & Chiu, 2004; Nicholson, Meyer, Flately, & Holman, 2013). This approach is shown to have an advanced impact on patient and caregiver interaction, health outcomes, and satisfaction with care (Ekman et al., 2011).

Other interactions are described to be less emotional and more concentrated on the job that has to be done. The last theme *losing one's place* refers to how receiving home nursing care, ironically, may involve being overlooked. The narratives show that the older persons find themselves placed in and reliant upon a system where conformity rules. The services offered are portioned out and bound to explicit procedures, and the participants explain how they try to adjust to and be available for receiving help whenever it suits the system. The experience of our informants is that healthcare services have been reduced. In the beginning of their illness trajectory, when physical and acute symptoms were more persistent, visits from the homecare nurses were more frequent. One derivation from this may be that the system addresses physical and well-defined needs more easily than psychosocial or existential needs. Other research as well has found that psychosocial needs in rural palliative care patients are poorly met (Butow et al., 2012). The psychosocial aspects of palliative care have also been highlighted as a particular struggle for district nurses (Walshe & Luker, 2010). Following another thread, the last theme also insinuates objectifying of the patient and hence a lack of person-centred care. Interestingly, Goodridge & Duggleby (2010) found that nurses felt that the rural context profoundly affected their ability to provide person-centred care, in both positive and negative ways. In their study, familiarity and a long-standing relationship with a patient were generally viewed as beneficial and contributed to a higher level of person-centred care. However, lack of anonymity could also lead to discomfort that in some cases could constrain closeness with patients and their families.

The strong place attachment demonstrated by the informants in this study suggests that the rural context may be an advantageous healthcare environment. Its potential to be a source of comfort, security, and identity concur with cancer patients’ strong desire to be seen as unique persons (Browall, Koinberg, Falk, & Wijk, 2012). Contrary to research findings that reflect nurses’ and other healthcare professionals’ perspectives, unfavourable aspects of the rural life are rarely voiced. The importance of remaining in one's home makes it worth picking up the pieces. Nursing in this context has a unique potential when it comes to personalized palliative care. However, the findings in this study suggest that nurses’ attitudes and involvement have the power to either emphasize or conceal the greatness in any “piece”.

## Conclusions and implications for nursing

Our results stress the importance of having the opportunity to remain in one's environment throughout the course of the illness, and of being surrounded by significant others.

The homecare setting, particular in a rural context, appears to be a favourable scene to allow for human individuality and originality to be displayed. Our study shows that district nurses play an essential role in the provision of palliative care for older rural patients. However, the therapeutic value of being in one's familiar landscape seems to depend on how homecare nurses manage to locate it and use it in a more or less person-centred manner. Provided that a stoic and modest attitude prospectively prevent expressions of unmet needs, communication skills and attentiveness to psychosocial aspects stand out as important attributes for nursing in this context.

## Methodological considerations

The life world of our informants is mediated through their narratives. A combination of phenomenological description and hermeneutic interpretation is required when achieving an understanding of human reality (Ricoeur, 1976). Ricoeur's interpretation theory offers a stepwise model presenting the analysis as transparently as possible. All three authors have reflected on and continuously and critically worked with the analysis until consensus was reached. The findings represent what we have found to be the most useful perspective when understanding the informants’ situations. Our interpretation have probably been influenced by our pre-understandings and professional experience (from palliative home nursing care/geriatric care/and mental health nursing). When considering the results, one must bear in mind the small number of informants and that they were relatively high functioning. Other meanings might have become more visible if worse physical health and symptoms had been present. On the other hand, they all knew that their time was limited, and several of them had died by the time this study was published.
